# Muslim considerations in seeking mental health help in California and Israel: a qualitative approach

**DOI:** 10.1186/s12889-025-22224-2

**Published:** 2025-03-20

**Authors:** Leena Badran, Niveen Rizkalla, Steven P. Segal

**Affiliations:** 1 School of Social Welfare, University of California Berkeley, Berkeley, USA; 2 Center for Effective Global Action (CEGA)and, the School of Public Healthaq, University of California Berkeley, Berkeley, USA; 3 Med School, Psychiatry and Behavioral Science, University of California Davis, 4701 X St, Davis, CA USA

**Keywords:** California, Considerations, Israel, Muslims, Qualitative Study, Seeking mental health help, Theory of Planned Behavior

## Abstract

**Background:**

Existing evidence indicates that Muslim minorities underutilize mental health services despite a pressing need. Employing the Theory of Planned Behavior (TPB), this study seeks to explore considerations that influence mental health help-seeking by Muslims residing in California and Israel.

**Methods:**

A qualitative approach involving semi-structured interviews guided by the TPB principles was implemented with 78 Muslim participants. Thematic analysis was conducted to identify key themes.

**Results:**

Employing both deductive and inductive approaches, four major themes were identified: attitudes (advantages, disadvantages, and the influence of religiosity), subjective norms (the impact of significant others), perceived behavioral control (facilitators and challenges), and intentions toward seeking mental health support (influenced by gender, and prior experience). Common social and cultural norms were identified in both groups within the patterns of the TPB. The family's significance as a supportive resource emerged in both groups, but the extended family had a more profound impact among Muslims in Israel. Stigma as a barrier against seeking mental health help was stronger among Muslims in Israel, while financial barriers and socio-political context were highlighted more by Californian Muslims.

**Conclusions:**

The findings highlighted the importance of adopting a holistic approach to mental health help-seeking among Muslims due to commonalities in approaches, irrespective of geographical differences. Variance between the two groups primarily stemmed from social factors, particularly stigma and the influence of extended family. The results underscore the universality of common aspects and emphasize the importance of addressing social norms and socio-economic realities to enhance engagement among Muslims in both countries.

**Supplementary Information:**

The online version contains supplementary material available at 10.1186/s12889-025-22224-2.

## Background

Muslims in the United States (U.S.) are hesitant to seek formal mental health care, despite the substantial need, leading to the underutilization of these services [1, 2]. The Muslim community in California faces a variety of social, cultural, and political challenges such as cultural adjustment struggles, immigration difficulties, and domestic violence—factors that are strongly linked to the emergence of mental health issues and heightened social stressors [3, 4]. These compounded social stressors create a pressing need for culturally informed interventions. To address this need, it is crucial to understand the reasons behind the reluctance to engage with formal mental health services, as studies consistently show a general hesitance among Muslims to pursue professional care [5–9].

A similar pattern is observed among Muslims in Israel, where despite an established wide array of general mental health services, significant underutilization persists [10]. A survey conducted by the Myers-Joint-Brookdale Institute [11] in Israel indicates that Arabic speakers have a lower prevalence of help-seeking from professional mental health services compared to informal assistance. Specifically, the percentage of Arabic speakers (16%), 22 years of age, who sought formal mental health services was notably lower than Hebrew speakers (54%), and Russian speakers (34%). Furthermore, a larger proportion of Arabic speakers (49%) exclusively sought mental health help from informal sources compared to Russian (41%) and Hebrew speakers (26%) [11]. In their study, Abo-Rass et al. [12] found that among 231 Palestinians living in Israel, attitudinal barriers significantly predicted the underutilization of professional mental health assistance. This underutilization of specialized mental health services highlights a substantial gap in the field of mental health and underscores the necessity for research exploration of its origins.

Seeking mental health help for emotional distress or psychological disorders is defined as an action performed by individuals to seek both formal (e.g., mental health professionals such as psychologists, therapists, or psychiatrists) and informal support (e.g., peer groups, friends, or family members) in the purpose of addressing mental health concerns [13]. When considering differences in service utilization and attitudes towards specialized mental health treatment among diverse ethno-racial and religious communities, studies have shown that certain minority groups (e.g., Black, Asian communities) and certain religious minorities (e.g., Muslims) tend to seek help for mental health problems less often than the general population [14–18]. Several factors impact the underutilization of formal mental health services among Muslim communities. Explanatory models in which mental health issues are attributed to supernatural causes (such as possession, the evil eye, or a test from God) often lead individuals to seek help initially and sometimes primarily from religious or spiritual leaders instead of mental health professionals [19]. Negative attitudes rooted in cultural beliefs and misconceptions toward mental health conditions, a lack of culturally sensitive services, and limited access to Muslim mental health professionals further discourage help-seeking. Additionally, the social stigma surrounding mental illness in Muslim communities creates fear of judgment and isolation, which in turn reinforces avoidance of formal care [9].

The political context, in which Muslim communities in both California and Israel operate, plays an additional role in shaping attitudes and help-seeking behaviors for mental health conditions. In California, Islamophobia—demonstrated through discriminatory policies, media bias, and social prejudice—creates an environmental factor of fear and mistrust [20]. Muslims in California represent a diverse population with varying sub-groups, migration histories, and levels of integration. Muslims who experience or witness incidents of Islamophobia may develop increased anxiety, depression, and stress, and often hesitate to seek mental health care due to concerns of being misunderstood, stigmatized, or judged [21]. This reluctance is further compounded by the lack of cultural humility perspective of the providers and fear of profiling or labeling within clinical settings, particularly for visibly religious individuals, such as Muslim women who wear the hijab [20].

In Israel, Muslims face unique socio-political challenges, that include systemic inequality, structural discrimination, and political conflict, which could have a profound impact on their mental health and willingness to seek care [22]. Ongoing stress and tensions together with the traumatic events contribute to unique and significant mental health needs. The complex socio-political reality in Israel shapes the subjective norms within the Muslim community, where seeking formal mental health care might be regarded with suspicion or as impractical [23]. Together, these socio-political factors could contribute to the systemic underutilization of formal mental health services in both the geographical contexts of California and Israel, which underscores the necessity for an in depth culturally adapted and politically informed interventions.

Even though timely interventions for mental health problems offer significant benefits, alleviate the suffering of affected individuals, and help prevent the progression of psychological issues into chronic mental illnesses [24], studies consistently reveal a gap between the need for specialized mental health services and their actual usage among minority groups [9, 25, 26].

**Fig. 1:**
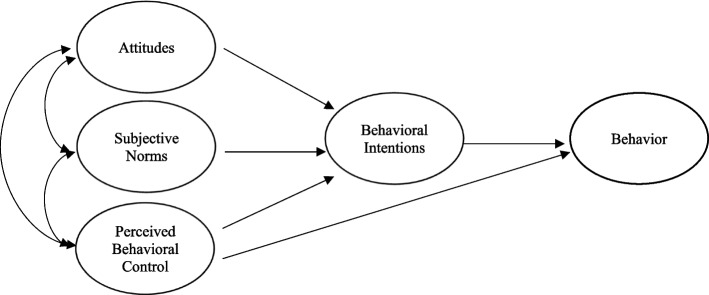
The Theory of Planned Behavior (TPB)

The Theory of Planned Behavior (TPB) [27, 28] highlights three cognitive components — attitudes, subjective norms, and perceived behavioral control — as crucial to explaining behavioral intentions, and subsequently, the behavior itself. A person’s attitudes toward a behavior reflects their personal predisposition toward engaging in the behavior. Subjective norms reflect an individual's understanding of social pressures, including the belief that significant others approve or disapprove of a behavior and their thoughts of his/her engagement in it. Perceived behavioral control, is a factor that accounts for the impact of personal abilities and external limitations. TPB posits that individuals are more inclined to perform a behavior if: (1) they hold positive attitudes towards it, (2) they perceive significant others, whose opinions they value, believe they should engage in the behavior, and (3) they feel they possess the requisite resources and opportunities to undertake the behavior (see Fig. 1).

This theory provides a framework for understanding factors in both the individual (e.g. attitudes and beliefs) and external social and environmental levels (e.g. socio-cultural norms, such as collectivist values, and the pivotal role of family and community) that have an impact on decision-making and help-seeking behavior. It is especially relevant given that mental health help-seeking is often influenced by social factors such as stigma, religious beliefs, and perceived control over health-related behaviors together with individual attitudes. TPB offers a framework for understanding these complex dynamics. Research has underscored the effectiveness of TPB in deciphering a range of health behaviors [29–32]. A few studies have considered intentions to seek help from mental health professionals among general medical practice patients [33] and college students [34]. Prior research highlights the significant role of religion (religiosity and attributions) [20] in seeking mental health help. To the best of our knowledge, no study has applied the TPB to consider seeking mental health help by Muslim minorities. This study aims to examine the social and structural considerations, aside from religious beliefs, which influence Muslims’ decisions to seek mental health services in California and Israel.

## Methods

### Participants and procedure

Seventy-eight Muslim participants, 47% from California and 53% from Israel, took part in this qualitative study. The majority of participants were women (58%), 69% were married, and had a moderate economic status (70%). The age range was 18–67 (M = 40, SD = 9.7). The sample included both mental health professionals (5%) and non-professionals from the general population. Among all participants, 39 identified as religious, 34 as traditional, and five as secular. Although 32% reported seeking professional mental help, only 15% had received a diagnosis of mental health issues. Notably, participants represented diverse geographic regions in both California and Israel (see Table 1).

**Table 1:** *Sample Characteristics N* = *78*

Participant’s group	California Israel	(N = 37)	(N = 41)
Gender	Men	10	10
	Women	27	31
Higher education	B.A	12	16
	M.A	9	9
	PhD	2	3
	Student	10	1
	Other	4	12
Marital status	Single	16	4
	Married	21	33
	Divorced	-	3
	Separated	-	1
Work in the mental health sector	Yes	2	18
	No	34	22
	Not sure	-	1
Level of religiosity	Very religious	6	3
	Religious	23	8
	Traditional	7	27
	Secular	1	3
Economic status	High	4	6
	Moderate	26	29
	Low	7	6
History of seeking mental help from a therapist	Yes	12	16
	No	24	25
	Not sure	1	-
History of psychiatric counseling	Yes	3	12
	No	34	29
Diagnosed with mental health issues^*^	Yes	4	8
	No	33	33
Residence status	Immigrants to U.S. (1st generation)	16	0
	Immigrants to U.S. (2nd generation)	21	0
	Indigenous Arabs in Israel	0	41
Age (years)		31 ± 13	42 ± 10

^*^Diagnosis of mental health issues included Schizophrenia, Anxiety, OCD, and PTSD

The study was approved by the Institutional Review Board of the University of California Berkeley (2022–12–15,929). In both California and Israel, the researchers (the first two authors) approached Muslim community centers, educational institutions (e.g., schools, universities), and various Muslim professional groups for the recruitment process. Locations of recruitment included Northern and Southern California, as well as Southern, Central, and Northern Israel. Initially, researchers approached Imams of six mosques, coordinators of projects, and other community leaders and four Muslim centers and sought their help in recruiting participants for the interviews. They also approached parent groups at schools, Muslim student groups, and other professional Muslim groups. When collaboration did not yield high responsiveness, snowball sampling was utilized as the recruitment method, in which participants who initially agreed to be interviewed were asked to assist in bringing or connecting the researchers with other potential participants. Inclusion criteria involved being a Muslim residing in California or Israel, at least 18 years old, and proficient in English or Arabic. Participant recruitment occurred between March and September 2023 and concluded upon reaching data saturation [35], indicating that additional interviews were unlikely to yield new information. Prior to participation, participants were sent an information sheet pertaining to the study’s purpose and procedures. Verbal consents were obtained after the first author explained to participants about data preservation and confidentiality. To protect the participants’ anonymity and confidentiality, participants agreed and suggested pseudonyms of their choice. All Muslim participants from Israel preferred to conduct the interviews in Arabic, as it is the native language of Muslim Arabs who are Israeli citizens, with English being their third language after Hebrew. In contrast, interviews with Californian Muslims were primarily conducted in English, except for three participants who preferred Arabic. All Muslim participants from Israel were Indigenous Arabs who were born in Israel and held Israeli citizenship, while the Californian Muslim participants represented a more diverse background: 16 were first-generation immigrants (foreign-born from regions including North Africa, South Asia, Europe, Southeast Asia, the Middle East, and West Africa), and 21 were second-generation individuals born in the U.S. to at least one immigrant parent. This diversity among the Californian participants likely reflects the extent of diversity and differences in acculturation levels and differing social norms around mental health, which can shape their attitudes, subjective norms, and perceived behavioral control regarding help-seeking. On the other hand, the homogeneity of the participants from Israel, combined with their shared socio-political context, provides a more unified and shared framework for understanding their mental health behaviors.

### Instrument

The co-authors include a Palestinian Muslim who has lived in the United States for the past four years and a Palestinian Christian, both of whom previously lived in Israel, and an American Jew who has conducted extensive research in Israel and its surrounding countries—all with prior experience researching mental health issues among minorities. The authors adjusted a series of 10 open-ended questions based on the guidelines of the TPB [36]. While the original guidelines include examples related to physical exercise, the study’s questions were adapted to focus on mental health help-seeking behaviors (please see the supplementary file for further details). The TPB instrument has been successfully used for mental health-related questions in previous research, though the specific questions were not provided in these studies [37]. The current study’s questions addressed three key areas: attitudes (advantages/disadvantages, four questions), subjective norms (people’s approval/disapproval, four questions), and behavioral control (two questions). The questions were specifically designed to examine the behavior of seeking mental health help, following Ajzen’s TPB guidelines [36].

#### Data analysis

Conducted in a semi-structured format, the interviews were carried out by the first author through approximately 90-min Zoom sessions in the participants' preference of time and language (English/Arabic). Verbal consent was obtained, and participants completed demographic surveys via a Qualtrics link before the interviews. The interviews were audio-recorded, transcribed, and translated for analysis. The two first authors independently coded all the transcripts. Subsequently, they compared their analyses and identified themes derived from participants' responses within the three domains of the Theory of Planned Behavior (TPB), employing a deductive thematic approach. Codes and themes were discussed and developed using Braun and Clarke’s [38] six-phase method for qualitative thematic analysis that includes familiarizing with the data, generating initial codes, searching for themes, reviewing themes, defining and naming themes, and writing the report. In the familiarization phase, the first author translated and transcribed the verbal data. During the initial coding phase, the first two authors read the translated transcriptions and generated initial codes by simplifying and structuring the data for theme identification. For example, one participant stated:

“Understanding Sakina (serenity) by itself helps at the end of the situation. I think it could be an advantage of seeking mental help, and just venting.”

This statement was initially coded as “religious understanding as coping,” “serenity (Sakina) linked to mental health improvement,” and “value of venting in seeking help.” The authors inductively identified major themes from these codes. In this case, the quote contributed to the major theme “Attitudes Toward Mental Health Help-Seeking” and the subtheme “Advantages of Improvement of Mental Health.” In the reviewing and refining phases, the first two authors separately audited the analysis by comparing the codes and themes with the original transcripts to ensure trustworthiness. Conflicting feedback was resolved through collaborative discussions among all authors until consensus was reached, ensuring that identified major themes and subthemes were evidenced in the transcripts.

This collaborative process led to a clearer definition of the themes and a more cohesive organization of both themes and subthemes, as well as the relationships between them. The third author supervised the study design, ethical and institutional procedures, the manuscript preparation, and reviewed and edited the final version of the manuscript.

## Results

Our analysis yielded four major themes with sub-themes. In this section, we delineate the similarities and differences between the groups when appropriate (Please see Table 2).

**Table 2:** Themes and sub-themes of mental health help-seeking in the study

**Major themes**	**Sub-themes**	
**Attitudes**	Advantages	• Improvement of Mental Health • Symptom Management • Guidance and Advice • Emotional Support • Prevention and Awareness
	Disadvantages	• Misdiagnosis or Ineffective Treatment • Social stigma and shame * • Dependency concerns on therapist and medication*
	Religiosity impact	
**Subjective norms**	Approval	Family members (parents, spouses, siblings, and children), close friends, coworkers, imams*, and their physicians
	Disapproval	Parents, spouse, extended family*, friends, in-laws
**Behavioral Control**	Facilitators	• Previous knowledge • Medical coverage* • Social support and assistance • Technological factors
	Challenges	• Logistics issues 1. Shortage in providers 2. Schedule an appointment 3. Time constraints • Financial barriers* • Stigma and cultural socialization * • Misconceptions of mental health issues • Confidentiality and privacy • Sociopolitical context
**Intentions**	Religiosity	
	Gender	
	Prior personal experience	

^*^Indicates major differences between both groups: Muslims in California vs. Muslims in Israel

### Attitudes

The attitudes delineated the advantages and disadvantages perceived by each participant when seeking mental help and the impact of religiosity. While both groups expressed several common advantages, significant disparities arose regarding the disadvantages.

#### Advantages

All participants from both groups expressed favorable attitudes toward seeking mental help and each participant addressed at least one positive attitude toward seeking mental help. Participants suggested the following benefits of seeking mental health support:

- *Improvement of Mental Health*: Participants mentioned that they found it helpful for venting and reducing stress. Nariman shared: “Understanding *Sakina* (serenity) by itself helps at the end of the situation. I think it could be an advantage of seeking mental help, and just venting” (woman, 19 years, CA).
- *Symptom Management*: Participants claimed that seeking mental help contributed to identifying the mental health condition they experienced, and thus were able to address the problem by starting with receiving a diagnosis for it.
  “I hadn't even told my mother or father, I never told anybody [...], finally I decided to get help, so it was kind of a big relief to say [...] okay this is, we identified it, and I can speak about it now” (man, 29 years, CA).
- Having a diagnosis of a mental health problem was received with relief, in having a name for the bothering symptoms.
  “If someone is experiencing unexplained physical symptoms like heart palpitations, and medical tests reveal no issues, consulting with a therapist can lead to a diagnosis of underlying anxiety, providing relief and a path toward treatment” (woman, 42 years, IL).
  *Guidance and Advice*: Most participants highlighted that therapists play a crucial role in helping individuals gain insights into their experiences, develop practical coping strategies to navigate their challenges, and access non-judgmental guidance. One participant said: “Therapists can offer valuable insights and coping strategies” (woman, 58 years, IL)
  Another participant emphasized the importance of receiving professional guidance. “I’m going to get guidance from someone who researched and analyzed human behavior and has seen multiple situations, and they would possibly be able to guide me a lot better than [my parents], the help that I need is a situation completely beyond my control” (woman, 44 years, CA).
- *Emotional Support*: Twenty (ten in each group) participants indicated that speaking with a mental health specialist provided emotional support, validation of their feelings, and reassurance that they weren't alone.
  “When I went to the psychiatrist follow-up meeting, I saw many people in the waiting room. This reassured me that I wasn't alone in seeking mental health support” (man, 67 years, IL).
- For many participants (N=12), regulating their emotions and stressful daily life was one of the goals that promoted support-seeking.
“I have some sort of process in which I could become a better person in terms of regulating my emotions better[...]. I also utilize mental health services to help navigate ambiguous situations like work situations [...]. It's really just going back to the point of life validation” (woman, 28 years, CA).
- *Prevention and Awareness*: Early intervention was noted to prevent mental health deterioration and lower the risk of long-term complications. One participant explained the importance of timing: “It’s also better to reach out when you're first feeling stressed, rather than later” (woman, 18 years, CA).

#### Disadvantages

While both groups displayed positive attitudes toward seeking mental help, participants from California demonstrated fewer perceived disadvantages compared to their counterparts, except in one sub-theme, in which both expressed the same degree of concern.

##### Stigmatization

Regarding social stigma and shame, two-thirds (66% N = 27 of 41) of the participants in Israel, in contrast to 50% (N = 19 of 37) of the Californian participants, identified stigma as a prominent disadvantage in contemplating mental health care. It was acknowledged as a significant barrier for individuals in need of assistance. One participant explained that stigmatization can potentially lead to adverse consequences, which might also contribute to perceptions of weakness or vulnerability:

“I also heard a lot about people who had a psychological file in their sick history that they fell into a lot of problems. I mean by that, for example, if a married woman was unsuccessful and asked for a divorce, we find many people using the mental health file under the pretext of taking away the mother from the custody of the children. This will prevent the guarantee of many rights, because I am the crazy one, and I am the one who has psychological problems. Despite that I will seek mental help but secretly” (woman, 45 years, CA).

##### Dependency Concerns on Therapists and Medications

Seventy-five percent of participants in Israel expressed concerns about medication dependency and side effects, whereas less than half of the Californians shared similar worries: “Finding the right medication can involve multiple trials, leading to uncomfortable side effects and dependency on it” (man, 47 years, IL). Certain participants found themselves handling such side effects: “Due to the side effects, I had to reduce my work, my work requires me to be alert most of the time because it requires extreme caution” (man, 67 years, IL).

##### Misdiagnosis or Ineffective Treatment

Participants from both groups doubted treatment effectiveness, especially when psychiatrists prioritized diagnoses and medications over understanding the patient. Therapy, though causing short-term discomfort, addressed emotionally challenging issues: “If you talk to the wrong person maybe if they're not qualified, they can make your situation worse or if you talk to the wrong doctor he will prescribe the medication that you will become addicted to that makes your situation worse” (man, 44 years, CA).

### Religiosity and attitudes

Our qualitative analysis explored the impact of religiosity on participants’ attitudes toward seeking mental health help in both groups. Participants who identified as religious or traditional were generally open to discussing mental health and seeking professional help. They often used religious teachings or statements as a reference that encourage Muslims to handle both physical and mental well-being equally, framing mental health conditions as part of normal life experiences. While these participants acknowledged the importance of professional care, they frequently relied on religious or spiritual coping strategies, attributing mental health conditions to the weakness of faith, evil eye, or other supernatural forces. As one woman said:

“As a religious woman who reads the Qur’an and believes in God Almighty, I initially sought to understand mental illness through a spiritual lens. I wondered if it could be caused by the evil eye, magic, or other spiritual factors. From the start, my focus was on uncovering the underlying cause of mental illness. If I feel psychological distress, I will definitely seek mental help as it is very beneficial and our religion, Islam, indicates that we are responsible for our general health” (woman, 58 years, IL).

In contrast, participants who identified as less religious primarily adopted a biomedical perspective, perceiving mental health as a clinical issue rather than one influenced by spiritual or moral factors. This group emphasized the role of psychological and medical interventions, with less reliance on religious explanations or practices.

“I picked secular and for me, it [mental health condition] is something biological that needs medical treatment. I prefer a psychiatrist to give me a pill and have immediate results” (man, 47 years, CA).

### Subjective norms

Subjective norm pertains to the opinions of influential members within a group, reflecting their endorsement or disapproval of a specific behavior. The social context for each group played a crucial role in shaping subjective norms. In both groups, family support emerged as a central element, while stigma served as a moderating factor. When stigma was pronounced, participants often faced disapproval from their extended families, particularly evident among Muslims in Israel compared to their counterparts. The latter group emphasized the significance of religious perspectives in seeking mental help, seeking approval from community religious figures as a crucial step.

#### Approval

Participants in both groups (more than 75% of each group) indicated that family members (parents, spouses, siblings, and children), close friends, coworkers, imams, and their physicians may approve of their pursuit of mental health assistance. Notably, Californians emphasized the importance of seeking preapproval from the imam, community figure, or religious leader more than their counterparts. “My wife is a nurse so she would be very supportive. My family, most of them are in healthcare so they understand” (man, 40 years, CA).

#### Disapproval

While certain individuals found support within their close circles for seeking help, others encountered resistance from family, spouses, or friends. Intriguingly, some women refrained from discussing their decisions or conflicts with in-laws due to cultural beliefs and apprehension of potential shame. In both groups, seeking help would likely face disapproval from the extended family, but its impact was more pronounced among participants in Israel, possibly due to living in geographic proximity.

“My father might disapprove and may advise against it. Additionally, I would avoid discussing my decision with my husband's family, particularly my father-in-law's household, as they might react negatively. They might question what happened to me and potentially label me as having gone ’crazy.’ This would put me in a vulnerable position” (woman, 42 years, IL).

### Behavioral control

Two sub-themes arose: facilitators; factors that promote help-seeking, and challenges; factors that inhibit help-seeking.

#### Facilitators

##### Previous Knowledge

It appears that both groups recognized the necessity of seeking mental health support based on their respective experiences. Participants' mental health knowledge came from professional backgrounds, personal experiences, and education, shaping their views on seeking care.

“Having a family member with mental health conditions and my work in the field, help a lot and make it easier […] to make an appointment soon” (woman, 45 years, CA).

*Social Support and Assistance:* For both groups, receiving assistance from families or friends, either through accompanying them to mental health settings/facilities, or offering recommendations for therapists, was noted by participants as greatly supportive.

“If there is a clear recommendation from someone, if there's an [experience], if someone has tried a psychologist and they told me; ‘if you go to this person, they are private, they're good,’ I think this would help” (woman, 36 years, CA).

##### Medical coverage

While the approach to delivering mental health services in Israel was perceived as beneficial, American Muslims (more than half) viewed the health system as a barrier. For participants from Israel, mental health care covered by the Israeli government was perceived as accessible without financial concerns, whereas this sub-theme was viewed as a barrier for Californians. One of the Californian participants said that due to her family’s socioeconomic status, they are not able to afford therapy sessions or medical coverage. As a result, they will prioritize physical health needs over mental health.

*“*I come from a low-income family so if these therapists are less expensive or just more available for people of lower income, I think that would be really helpful, because if I wanted to seek help but found out that it was too much money, I don't think I would get it, because as much as I want to prioritize my mental health there are a lot of other expenses and things that we (my family) have to think about. So, for me, I would put my problems aside just because they wouldn't be able to spend that much money on getting help” (woman, 19 years, CA)

“I don't need to pay a substantial amount for each psychiatric session as it is covered by the Israeli government” (man, 63 years, IL).

##### Technological Factors

Technology facilitated participants' access to mental health support through remote platforms, eliminated the need for transportation efforts and costs, and allowed greater flexibility for scheduling appointments for Californians Muslims more than Muslims in Israel.

“Now we live in a telemedicine world, so if I can, I would prefer to have these conversations virtually that would work within my schedule versus going to see them and book an appointment and spending like the whole day wasted to just go there for that” (man, 32 years, CA).

#### Challenge

##### Logistics issues

***Schedule an Appointment:*** Certain bureaucratic procedures posed some challenges with appointment scheduling.

“It's difficult to set up the appointments; sometimes the professionals are really booked, so you have to wait a few months for an appointment, and then when those months come by, the stress you feel is a little bit less, and you kind of move past that part of your life” (woman, 18 years, CA).

***Time Constraints*:** Balancing work, family, and other responsibilities made it difficult for participants to find the time to attend therapy sessions or seek treatment.

“I’m a mother of two daughters, and their needs often take precedence over mine, a common situation among Arab women due to societal expectations” (woman, 34 years, IL).

***Shortage in Providers*:** In certain geographical areas in both California and Israel, there might be a shortage of mental health professionals for in-person sessions, resulting in extended waiting times or lists and a limited choice of providers, thereby reducing access to essential support. However, this issue was emphasized more among Californians.

“It's difficult to set up the appointments like sometimes the professionals are like really booked so you have to wait a few months for an appointment and then when those months come by like the stress your feeling is like a little bit less and you kind of move past that part of your life” (woman, 19 years, CA).

The shortage of providers was further emphasized by the challenge of finding a Muslim therapist who understood and was familiar with the social and cultural norms.

“I don't feel that there are enough Muslim mental health specialists, so that is really scary to me, because I might be coming from a perspective, but this non-Muslim mental health specialist will be like ‘you don't have to worry about that, it shouldn't be a problem.’. But it is a problem in my eyes” (woman, 18 years, CA).

***Misconceptions of Mental Health Issues:*** Participants stated that misconceptions and misunderstandings of mental health issues are a significant barrier to seeking mental support.

“In our Arab and Muslim society, people often blame the individual, saying, ‘You have a problem; you can't handle it; you're tired.’ Instead of recognizing stress as a common challenge, they accuse the person of not knowing how to manage life” (woman, 39 years, CA).

***Confidentiality and Privacy:*** Confidentiality and privacy concerns were reported by participants from both groups as being significant barriers to preventing help-seeking.

“I'm not sure how comfortable I would be sharing with a mental health specialist about my challenges” (man, 32 years, CA).

***Financial Barriers:*** Mental health care can be expensive, even with insurance. High co-pays, deductibles, or limited coverage created financial barriers to accessing treatment, especially among Californians. Thus, both groups deprioritized seeking mental help.

“If seeking help becomes too expensive, I might prioritize other expenses over my mental health, as the financial burden could deter me from getting the support I need” (woman, 18 years, CA).

***Stigma and Cultural Socialization:*** Despite the efforts to normalize mental health support, participants still articulated significant social stigma surrounding mental health issues and seeking help among the Muslim communities. Stigma was an enhanced concern among the participants from Israel due to their social lifestyle and geographic proximity to their kin.

“The Muslim community has a large stigma against mental health and treatment in general because a lot of people are labeled ‘crazy’. Someone who wants to get treatment [...], that type of talk demotivates people from getting the help that they need” (woman, 18 years, IL).

***Sociopolitical context:*** For Muslim responders in California (N = 10, 27%), Islamophobia was experienced as a political barrier, manifest in political and social contexts, where political discourse, media representation, and policy impact Muslims’ everyday interactions. As one participant noted:

“Islamophobia has a serious impact. As a Muslim, wherever you go, you will get identified as a Muslim; inside the airport, when you get pulled over, or sometimes when you have been with a doctor, and he reads your name. He will know that you are a Muslim. But sometimes you find differences in how you are treated; I think being a Muslim is a barrier to receiving some services and even to being willing to receive some services. And I will keep this in the back of my mind every time” (man, 47 years, CA).

In Israel, fifteen participants (36%) stated that the political climate and the ongoing Israeli-Palestinian conflict at times heightens feelings of mistrust and division between Muslim and Jewish communities, influencing mental health help-seeking preferences.

“In times involving political conflict, I prefer to avoid seeking any kind of physical or mental help from the Jewish community” (woman, 48 years, IL).

### Intentions

Religiosity, gender, and prior experience with mental health care have significantly influenced participants’ intentions toward seeking mental help. Religious participants expressed a preference for initially consulting religious figures and utilizing faith-based coping mechanisms.

“For me and my extended family, it is ok to consult with imam at the beginning, this may bring some comfort, and I don’t think that this is a bad thing” (man, 40 years, CA).

In contrast, less religious participants leaned toward a biomedical approach, favoring medication as a primary means for faster results.

“If my friend has a mental health issue, I will definitely just suggest to them to seek mental health help from a professional, to help them out first” (man, 55 years, CA).

Both men and women exhibited hesitation in seeking mental health help due to social and cultural norms. Men feared being perceived as weak or mentally soft, while women were concerned about potential harm to their family’s reputation or future marriage prospects.

“I would think carefully before seeking formal help, as being a man, it could negatively affect my social image” (man, 25 years, CA).

Participants with prior exposure to mental health care, whether through personal experience, family members, or professional settings, demonstrated a greater willingness to seek mental health support when needed, indicating that familiarity with mental health services played a key role in reducing hesitation. As one participant said:

“My cousin has a mental health condition, and it took her family a long time to seek professional help for her. Now, I can see the lasting impact of that delay. Because of this experience, I would be more encouraged to seek mental health help if I ever needed it” (woman, 41 years, IL).

## Discussion

The present study utilized the TPB to examine mental health help-seeking behavior among Muslims in California and Israel. Our analysis identified several major themes, including attitudes (advantages, disadvantages, and the influence of religiosity), subjective norms (the impact of significant others), perceived behavioral control (facilitators and challenges), and intentions toward seeking mental health support (influenced by gender, and prior experience). This study offers a unique perspective of exploring how similar social-cultural and religious factors shape mental health help-seeking behavior in two Muslim minority populations situated in distinct geographic and socio-political contexts. While many studies have focused solely on negative attitudes and barriers toward seeking mental help, our study delved into both positive and negative attitudes towards seeking mental health support within Muslim communities, alongside exploring the facilitators and barriers that better represent the experiences of Muslim communities when seeking mental health assistance. A deductive approach was employed to analyze participants' responses, resulting in the identification of four major themes and sub-themes.

### Attitudes

The study findings show a general shift in the Muslim population towards more positive attitudes, replacing the long-standing negative perceptions that have historically framed mental health as a social taboo. The data indicated that Muslims across the two groups shared detailed attitudes toward seeking mental health assistance, acknowledging both its potential benefits and drawbacks. Participants highlighted several positive aspects of seeking mental health help, such as improving overall well-being, managing symptoms, offering emotional support, and providing guidance. These perceptions pointed to an underlying awareness of the potential value of mental health services when they were perceived as effective and appropriate. Such positive attitudes have been indicated in other studies, which similarly found that despite barriers, Muslims may seek formal care when they believe it will yield tangible benefits [9, 39]. In our study, religion was seen as a factor that encouraged seeking mental health support. Religious teachings emphasized the importance of caring for both physical and mental well-being, framing them as the individual's responsibility. This finding aligns with previous research highlighting the positive role of religion in promoting well-being and encouraging individuals to seek mental health support [40, 41].

However, participants also expressed significant concerns, including social stigma, fears of dependency on therapy or medications, and apprehensions about potential misdiagnosis or ineffective treatment. The fear of becoming dependent on therapy and medications reflects anxieties surrounding long-term reliance on external assistance. This concern may stem from cultural values that emphasize resilience, and reliance on informal support networks, such as family or religious leaders. Additionally, participants’ worries about misdiagnosis and ineffective treatment point to a broader mistrust of healthcare systems [17]. This mistrust may be linked to experiences with culturally insensitive care or providers’ limited understanding of religious and cultural contexts [23].

Social stigma was also articulated as a pervasive issue in both Muslim communities because mental illness was often stigmatized, and seeking professional help could be viewed as a source of shame or weakness. This concern about stigma aligns with other studies showing that fear of negative judgment from family and community members deters many Muslims from accessing mental health care [19, 20]. Stigma may further exacerbate feelings of isolation for individuals struggling with mental health issues, making it less likely for them to engage with formal support systems.

### Subjective norms

Divergent responses in both groups were observed regarding subjective norms, with the majority perceiving family and parents as supportive factors, whereas some participants perceived them as unsupportive. These findings align with a broader body of literature on social support, where family and friends play a significant role in providing assistance with education, recommendations, and companionship [42, 43]. This role emphasizes the importance of a holistic approach to understanding mental health conditions within Muslim communities in both studied geographical locations. Participants emphasized that the approval or disapproval from their families played a pivotal role in shaping their intentions and decisions regarding seeking mental health care. This highlights that collectivist values still persist across different Muslim residential settings, where the family and community serve as central figures in influencing mental health decisions. For Muslims, support networks are often built around families, friends, religious leaders, and community members, who provide comfort and assistance that align with social, cultural, and religious values [20]. Regardless of geographical differences, social support systems remain a protective factor, offering emotional and psychological reinforcement. Both groups in our study reside in different locations and environments, yet some similarities were observed particularly in social norms and support. Despite most American Muslims in our study immigrating to the US, they continue to uphold their cultural norms, which influence their decisions and lifestyles. Despite undergoing an acculturation process and being geographically distant from their families, they still prioritize obtaining approval from their families. Furthermore, community-based and religious support networks, which are embedded in the socio-cultural fabric of Muslim communities, provide culturally relevant and effective alternatives to formal mental health services [43]. These systems, often more accessible and trusted, are acknowledged as integral components in mental health care, with the approval of family and community members serving as a powerful catalyst in seeking help. An intriguing finding regarding the disparities was the significance of seeking approval from religious figures among American Muslims compared to their counterparts. One possible explanation for this is that Muslims in Israel reside in towns where the population is predominantly Muslim, and religious aspects are integral to their daily lives. Consequently, religious figures are more accessible, and individuals can easily meet with them in their neighborhoods. Conversely, American Muslims live in different circumstances where they primarily encounter other Muslims during Friday sermons rather than in their immediate neighborhoods. Thus, seeking such approval demands effort and special consideration. This behavior serves as a means for them to maintain ties and connections with their religion and heritage. According to acculturation theories, such behavior may be described as falling between integration or separation, wherein individuals retain their cultural heritage [44]. Gender dynamics were influenced by social norms related to seeking help, in which men were more stigmatized when getting mental health treatment [45]. This stigma influenced kin approval, with Muslim women often managing mental health issues internally within their families. Many married Muslim women expressed reluctance to share their decision with in-laws, fearing judgment [17].

### Behavioral control

Results in perceived behavioral control factors, such as prior knowledge, insurance, social support [46], and technological aspects, were identified as facilitators. However, similar to the general population, participants in this study noted obstacles more than facilitators, including logistical issues [47] financial barriers [48], shortage in providers [49], and cultural stigma [50, 51]. Our findings align with previous studies indicating that stigmatization, shame, and economic challenges predict reduced intent to access psychological services among Muslims [52, 18].

The comparative approach used in this study revealed notable differences between Muslims in California and Israel, despite their similarities. Californian Muslims emphasized the scarcity of Muslim specialists more than their counterparts in Israel. This disparity may be attributed to two factors: first, the greater diversity within the Muslim community in California, consisting of immigrants from various countries with diverse cultural norms, different languages, and levels of religious adherence, compared to Muslims in Israel, who generally share the same language, cultural norms, and religious beliefs. Consequently, Muslims in California sought therapists who could cater to their varied backgrounds [52], whereas Muslims in Israel required fewer specific criteria when seeking mental help. Second, the availability of a wider variety of therapists in Israel who speak Arabic and share Muslim religious beliefs that align with participants’ social and cultural norms, made access more encouraging. However, this also raised concerns about confidentiality, as there was a higher likelihood of knowing the therapist from mutual social circles [53, 54]. The challenge of scheduling appointments underscores not only the importance of timely care but also reveals a common trend in both Muslim communities [55].

The political climate doesn’t prevent people from seeking treatment but adds to the challenges Muslims face. For many Muslims in the U.S., the concerns about seeking treatment arise from their physical appearance—such as wearing religious clothing, having distinct skin tones or Muslim names—which can make them vulnerable to prejudice, discrimination, or social stigma known as Islamophobia—a fear or prejudice against Muslims based on their religious affiliation [56, 57]. These concerns are compounded by the fact that Muslims in the U.S. live dispersed among diverse communities, with limited access to culturally familiar care. Consequently, they often have no alternative but to rely on public health services, in which they might feel isolated or misunderstood. In contrast, most Muslims in Israel live in more homogeneous, segregated communities, which makes it easier for them to seek care from professionals within their communities, either in their own towns or nearby Arab towns, where they feel culturally and linguistically supported. Yet, political conflicts and associated social stressors might affect their decision-making and attempts to initiate mental health care. These heightened levels of discrimination negatively influence Muslims’ attitudes toward seeking formal mental health care [58]. This underscores the need for greater awareness among therapists to better understand and accommodate the specific needs of Muslim clients.

Furthermore, Israel is smaller than the state of California. While Muslims in California, especially those in rural areas, may need to travel considerable distances for mental health services, individuals in Israel typically do not encounter such extensive travel demands. As a result, participants from California indicated a preference for remote therapy compared to their counterparts in Israel. This preference underscores the more stressful lifestyle experienced by Muslims in California, despite technological advancements and system development, highlighting the importance of efficient time management when seeking help.

Medical insurance and coverage for mental health care are different in the US system compared to the medical care system in Israel. The Israeli governmental fund covers all physical health services and mainly subsidizes mental health services expenses for Israeli citizens, including Muslims [59]. This reality encourages help-seeking behaviors since mental care costs are affordable, alleviating financial burdens during the process, unlike their counterparts in California. However, it's worth noting that despite mental health services being covered by the Israeli government, Muslims there still identify the financial factor as a barrier, particularly because many of them reside in under-resourced towns.

Social stigma and shame pose significant barriers to seeking mental help in both groups, with a more pronounced impact among Muslims in Israel. The social and geographical distance from extended family, relatives, and the collectivist community, with its traditional values, somewhat liberates Californian Muslims from the stress of social stigma when seeking help. They may feel less pressured by close circles’ approval or disapproval of their help-seeking intentions. In contrast, Muslims in Israel live in close proximity to their kin and communities, maintaining a conservative lifestyle and communal values where collective interests take precedence over individualistic ones. This intense living proximity in Israel makes seeking mental health support more intimidating, as individuals are compelled to conform to social norms and customs due to the visibility of their actions to everyone around them and the threat of exposure to judgment.

### Intentions

Gender was also found to be an element that may influence intentions toward seeking mental health help among both studied groups. Due to societal expectations, men generally hesitate to seek mental health support, fearing the perception of being "mentally soft" in a culture that values physical and mental strength as essential masculine traits [19]. Similarly, women also expressed reluctance to seek help, due to concerns about potential stigma [60] that can impact future opportunities, such as marriage prospects, career advancement, and parental rights such as child custody. Muslims often turn to religious leaders as their first point of contact to navigate the complexities and challenges associated with the social stigma of seeking professional help, with religious beliefs serving as a preferred coping mechanism. In addition, guidance from religious leaders can enhance the acceptance of formal mental health care by reassuring individuals that it is acceptable and compatible with their faith. Moreover, many Muslims rely on religious practices—such as prayer, scripture recitation, and involvement in faith-based communities—as a means of coping with the challenges caused by mental health issues. Research has shown that religious coping is strongly linked to social support, although it extends beyond this aspect solely [61]. Additionally, studies indicate that religion plays a significant role in shaping individuals' approaches to mental health.

### Limitations and future implications

Muslim groups in California are diverse and have unique and distinct cultural norms, even though they share a common religious identity. This diversity can make it difficult to generalize about the entire Muslim population in the U.S. One limitation of this study is the variation in the level of acculturation and exposure to Western mental health care among Californian Muslim populations. The Muslim participants come from various geographical countries of origin, but were mainly first-and second-generation immigrants, each with different degrees of adaptation to Western norms and culture, and openness to receiving mental health treatment when needed. This generational gap may affect how participants respond to receiving treatment, levels of exposure to Western norms, and accessibility to mental health services.

The Muslim participants in Israel were Israeli citizens. Therefore, the findings should not be generalized to Muslims living in the West Bank or Gaza or to other Muslim populations not included in our sample [62]. Additionally, most Muslims in Israel reside in separate towns, which limits their mobility and integration into the wider society. It may also limit access to public mental health services. In contrast, Muslims in California typically do not live in such segregated communities, allowing for greater exposure and access to mental health resources.

The TPB might not fully capture the diverse beliefs and practices among Muslim participants, as attitudes toward mental health and religious practices can differ significantly within Muslim communities, especially across various cultural and geographical settings. For instance, some participants may see mental health purely as a medical issue, while others may attribute it to spiritual factors, and some may combine both viewpoints. In all three scenarios, individuals could exhibit a variety of behaviors while holding multiple, sometimes contradictory, beliefs. The TPB may not sufficiently address this complexity.

At the theoretical level, utilizing the TPB allows researchers to comprehend the three patterns toward seeking mental help: positive and negative attitudes, subjective norms, and perceived behavior control. These results can apply to other Muslim groups across various locations and contribute to developing an adapted TPB questionnaire.

On a practical level, the significance of social groups as a crucial factor in encouraging help-seeking is evident. Practitioners in the community may design interventions involving family members and other community figures, incorporating them into the decision-making process as supportive factors. This approach aims to enhance mental health support within the Muslim community. For policymakers, it calls for work on raising awareness and developing more adapted and accessible mental health services tailored to Muslims.

## Conclusions

While both groups live in two different geographical regions, the findings highlighted commonalities in the approaches to seeking mental health help among Muslims. While most studies emphasize negative attitudes toward seeking help, it is crucial to also examine positive attitudes and to continue working to translate these attitudes into actions. Social determinants, particularly stigma and the influence of extended family, were common challenges among both groups, albeit with varying degrees of impact. The results underscore the universality of common aspects and emphasize the importance of addressing social norms, religion, and socio-economic realities to enhance engagement among Muslims in both countries.

## Supplementary Information

Supplementary Material 1.

## Data Availability

The datasets generated and/or analysed during the current study are not publicly available due to concerns of privacy and confidentiality, but some material can be available from the corresponding author on reasonable request.

## Abbreviation

TPB: Theory of Planned Behavior
