# Supplementary material for: Muslim considerations in seeking mental health help in California and Israel: a qualitative approach
Source: BMC Public Health. 2025 Mar 20;25:1077. doi: 10.1186/s12889-025-22224-2 (PMC11927169; doi:10.1186/s12889-025-22224-2)
Supplement: Supplementary file 1 — Supplementary Material 1. [file 12889_2025_22224_MOESM1_ESM.docx]

**Appendix 1**

**Interview questions following the Theory of Planned Behavior guidelines**

*Attitudes: Behavioral outcomes and experiences*

(1) What do you see as the advantages of seeking professional help such as counseling, psychotherapy, and psychiatric treatment when one is emotionally overwhelmed by making a phone call *or schedule an appointment*?

(2) What do you see as the disadvantages of seeking professional help such as counseling, psychotherapy, and psychiatric treatment when one is emotionally overwhelmed by making a phone call *or schedule an appointment*?

(3) What positive feelings do you associate with professional help such as counseling, psychotherapy, and psychiatric treatment when one is emotionally overwhelmed by making a phone call *or schedule an appointment*?

(4) What negative feelings do you associate with seeking professional help such as counseling, psychotherapy, and psychiatric treatment when one is emotionally overwhelmed by making a phone call *or schedule an appointment*?

Subjective Norms: *Normative referents*

When it comes to your seeking professional help such as counseling, psychotherapy, and psychiatric treatment when one is emotionally overwhelmed by making a phone call *or schedule an appointment*, there might be individuals or groups who would think you should or should not perform this behavior.

(1) Please list the individuals or groups who would approve or think you should seek professional help such as counseling, psychotherapy, and psychiatric treatment when one is emotionally overwhelmed by making a phone call *or schedule an appointment*.

(2) Please list the individuals or groups who would disapprove or think you should not seeking professional help such as counseling, psychotherapy, and psychiatric treatment when one is emotionally overwhelmed by making a phone call *or schedule an appointment*.

(3) Sometimes, when we are not sure what to do, we look to see what others are doing. Please list the individuals or groups who, after an emotional distressed event, are most likely to seek professional help such as counseling, psychotherapy, and psychiatric treatment when one is emotionally overwhelmed by making a phone call *or schedule an appointment*.

(4) Please list the individuals or groups who, after an emotional distressed event, are least likely to seek professional help such as counseling, psychotherapy, and psychiatric treatment when one is emotionally overwhelmed by making a phone call *or schedule an appointment*.

Behavioral Control: *Control factors*

(1) Please list any factors or circumstances that would make it easy or enable you to seek professional help such as counseling, psychotherapy, and psychiatric treatment when one is emotionally overwhelmed by making a phone call *or schedule an appointment*

(2) Please list any factors or circumstances that would make it difficult or prevent you from professional help such as counseling, psychotherapy, and psychiatric treatment when one is emotionally overwhelmed by making a phone call *or schedule an appointment*.
